# Heterologous signal peptide grafting enhances the immune efficacy of *Salmonella* vectors delivering hemagglutinin against H7N9 avian influenza virus

**DOI:** 10.1186/s13567-025-01590-0

**Published:** 2025-07-21

**Authors:** Wangyangji Sun, Rui Zhu, Yu-an Li, Zewei Li, Yuanzhao Du, Shifeng Wang, Huoying Shi

**Affiliations:** 1 College of Veterinary Medicine, Yangzhou University, Yangzhou, 225009 Jiangsu China; 2 Jiangsu Co-Innovation Center for the Prevention and Control of Important Animal Infectious Diseases and Zoonoses, Yangzhou, China; 3 Yebio Bioengineering Co., Ltd of Qingdao, Qingdao, 266114 China; 4 Department of Infectious Diseases and Immunology, College of Veterinary Medicine, University of Florida, Gainesville, FL 32611-0880 USA; 5 Joint International Research Laboratory of Agriculture and Agri-Product Safety, Yangzhou University (JIRLAAPS), Yangzhou, China

**Keywords:** Influenza virus, *Salmonella* vector, H7N9 avian influenza virus, tissue plasminogen activator signal sequence

## Abstract

*Salmonella enterica* serovar Typhimurium (*S*. Typhimurium) vectors, which induce broad cellular and humoral immune responses, are excellent candidates for delivering foreign antigens. However, *S.* Typhimurium strains display limitations, including low levels of antigen protein expression when delivering viral antigens. In this study, we found that replacing the hemagglutinin (HA) precursor sequence of H9N2 AIV (avian influenza virus) with that from H7N9 AIV significantly improved HA protein expression. Building on this, we combined the H9N2 HA leader sequence with a tissue plasminogen activator (tPA) signal peptide and delayed lysis *Salmonella* mRNA interferase regulation vector (SIRV) system previously developed by our team. This novel approach markedly enhanced the expression of viral antigens delivered by *Salmonella* vectors. Our results demonstrate that both the H9N2 HA leader sequence and the tissue plasminogen activator (tPA) signal peptide significantly increased H7N9 AIV HA protein expression and substantially improved the protective efficacy of the attenuated *S.* Typhimurium vector delivering the H7N9 HA protein vaccine against H7N9 AIV challenge. These findings offer valuable insights for developing more effective attenuated *Salmonella-*based recombinant H7N9 AIV vaccines and provide a valuable reference for vaccine strategies against other infectious diseases.

## Introduction

The prevalence of highly pathogenic avian influenza virus (AIVs) in poultry is associated with high mortality, posing a significant threat to the poultry industry and resulting in substantial economic losses [1, 2]. In recent years, antigenic drift has allowed these AIVs to become more susceptible to mammals, raising significant concerns for human health [2–4]. Although various vaccines targeting AIVs have been developed [3–11], traditional inactivated vaccines or subunit vaccines have been found to perform poorly in eliciting cellular and mucosal immunity, offering limited protection for the prevention and control of infectious diseases [12]. Consequently, there is an urgent need to develop novel, more effective vaccines to curb the spread of AIVs.

DNA vaccines have been proposed as a potential solution to poultry diseases due to their efficacy, affordability, and ease of construction [13–16]. However, their inability to induce strong immune responses and their current impracticality for large-scale use limit their application in the poultry industry [17]. Signal peptides are critical for efficient transcription and for directing the intracellular processing of newly synthesised proteins. [18, 19]. The tissue plasminogen activator (tPA) signal sequence effectively facilitates transport of proteins from the endoplasmic reticulum to the Golgi apparatus, thereby increasing antigen expression and secretion [18]. Modifying DNA vaccines with the tPA signal sequence has been shown to enhance the antigen expression and secretion [20]. Recently, we found that a signal peptide derived from the H9N2 AIV HA protein, when fused to the hemagglutinin (HA) protein of H7N9 AIV, markedly increased HA protein expression compared to the native signal peptide of H7N9 AIV (data not yet published). Thus, both the tPA signal sequence and the H9N2-derived HA signal peptide have the potential to enhance the expression efficiency of heterologous HA proteins through distinct mechanisms.

DNA vaccines require an efficient intracellular delivery system to maximise their efficacy by ensuring the effective entry of DNA into cells [21]. The intracellular nature of *Salmonella*, along with its highly engineerable characteristics, makes it an attractive platform for plasmid delivery intracellularly [22]. In various animal models, including mice and rainbow trout, attenuated *Salmonella enterica* serovar Typhimurium (*S.* Typhimurium) strains have been successfully engineered to carry plasmid DNA encoding pathogen-specific antigens, thereby eliciting robust humoral and cellular immune responses [23, 24]. In our previous work, we successfully used *S.* Typhimurium to deliver plasmids expressing the S1 protein of infectious bronchitis virus (IBV) and the HA protein of H9N2 avian influenza virus, both of which induced robust protective immunity [25]. Unlike vaccines delivered via extraintestinal routes, orally administered *Salmonella* can actively invade and colonise the host lymphatic system, activating the innate immune system to provide adjuvant properties [22, 26]. *Salmonella*-based vaccines can also induce mucosal and cellular immune responses against antigens and are cost-effective to produce, eliminating the need for needles and syringes during immunisation, thus offering economic benefits [26–29]. However, exogenous cargo carried by *Salmonella* vectors is often restricted by the bacterium’s membrane structures and the *Salmonella*-containing vacuoles (SCVs) it recruits, which limits access to the host cell’s cytoplasm [30–32]. Previously, we developed a *Salmonella* mRNA interferase regulation vector (SIRV) system [33]. This system enables *Salmonella* vectors to escape SCVs and actively release intracellular cargo by differentially regulating expression of the lytic toxin MazF in vivo and in vitro [33].

In this study, we integrated the H9N2 AIV HA protein signal peptide, the tPA signal sequence, and the SIRV system into a novel *S.* Typhimurium vector. We then assessed the expression levels of heterologous antigens, the immunogenicity of the vector, and its protective efficacy against AIVs.

## Materials and methods

### Animal and ethical statement

Specific pathogen-free (SPF) chickens were purchased from Boehringer (Beijing, China). All animals were housed in conventional animal facilities with ad libitum access to water and feed, and were monitored at least twice daily. All studies involving animals at Yangzhou University were approved by the Jiangsu Provincial Experimental Animal Management Committee (licence numbers SYXK (SU) 2021‐0027, SYXK (SU) 2017‐0044, SYXK (SU) 2021‐0026). These procedures conformed to international regulations as to Jiangsu Province’s ethical and welfare standards for the use of experimental animals.

### The construction of expression plasmids optimised for codon usage of HA

We extracted the signal peptide sequence from the *HA* gene of H9N2 AIV (WJ57) (Table 1), replaced the 1–48 bp sequence of the *HA* gene of H7N9 AIV (QHD1) (Table 1) with this sequence, naming the construct 9S7M. The 9S7M, full-length H7 HA coding sequence (CDS), and H7 HA non-coding regions (NCR; including the CDS and NCR regions of H7 HA) were then sent to GenScript for avian-preferred codon optimisation and gene synthesis. Kpn I and *Xho I* restriction endonuclease sites were introduced upstream of the start codon and downstream of the stop codon, respectively, to facilitate subcloning. The codon-optimised *HA* genes from these three versions were separately subcloned into the Kpn I and Xho I sites of the pS0017 vector, generating three eukaryotic expression plasmids: pS0017-9S7M, pS0017-CDS, and pS0017-2NCR (Table 1) [34]. Each eukaryotic expression plasmid was extracted from *E. coli* (χ6212 strain) (Table 1) using an endotoxin-free plasmid extraction kit (Tiangen, Beijing, China) for subsequent in vitro transfection.

**Table 1:** **Strains and plasmids used in this study**

Strain	Description	References
AIVs		
H7N9	A/Chicken/China/QHD1/2019(H7N9)	provided by Yebio Bioengineering Co., Ltd of Qingdao, China
H9N2	A/Chicken/Jiangsu/WJ57/2012(H9N2)	
*Salmonella*		
rSC0130	Δ*relA*::*araC* P_araBAD_ lacI TT, Δ*pmi*, Δ*endA*::TT *araC* P_araBAD_ *mazE* TT, Δ*cysG*:P_lac_ *mazF*, Δ*asdA33*	Lab stock
rSC0130(pS0017-9S7M)	rSC0130 containing eukaryotic expression plasmid pS0017-9S7M	This study
rSC0130(pS0017-CDS)	rSC0130 containing eukaryotic expression plasmid pS0017-CDS	
rSC0130(pS0017-2NCR)	rSC0130 containing eukaryotic expression plasmid pS0017-2NCR	
rSC0130(pS0017)	rSC0130 containing eukaryotic expression plasmid pS0017	
rSC0130(pS0017-tPA-9S7M)	rSC0130 containing eukaryotic expression plasmid pS0017-tPA-9S7M	
*E. coli*		
χ6212	Ф80d *lacZ*Δ*M15 deoR* Δ(*lacZYA-argF*)-*U169 glnV44* λ^−^ *gyrA96 recA1 endA1* Δ*asdA4* Δ*zhf-2*∷Tn*10 hsdR17* (R^−^M^+^)	provided by Dr Roy Curtiss III [22]
Plasmid		
pS0017	eukaryotic expression plasmid derived from the pcDNA3.1 backbone containing the cytomegalovirus (CMV) promoter, the asdA + balanced lethal system, and the Arabidopsis sugar-regulated mazE/F gene expression	Lab stock
pS0017-9S7M	pS0017 vector containing recombinant H7 HA with the H9 HA leader sequence replacing the 1–48 bp leader sequence of H7 HA	This study
pS0017-CDS	pS0017 vector containing the coding region of H7 HA from A/chicken/China/QHD1/2019(H7N9)	
pS0017-2NCR	pS0017 vector containing the coding region of H7 HA and 5'and 3'non-coding regions from A/chicken/China/QHD1/2019(H7N9)	
pS0017-tPA-9S7M	pS0017 vector containing the coding region of tPA sequence and 9S7M fragment	

Based on 9S7M, the tPA-9S7M recombinant *HA* gene was constructed by introducing the tPA signal sequence at the 5'end of the 9S7M fragment. The methods used to generate pS0017-tPA-9S7M were the same as those described above.

### HA protein analysis by western blot

In vitro expression of the HA antigen protein, using imprinting analysis, was conducted to validate the transient expression of HA antigens from various HA eukaryotic vectors. HA plasmids were first transfected into human embryonic kidney 293 T (HEK293T) cells (maintained in our laboratory) using PolyFect^®^ Transfection Reagent (Univ, Shanghai, China). In brief, 1000 ng of plasmid DNA was transfected into 80% confluent HEK293T cells in 24-well plates, and cell lysates were harvested after 48 h. Equal amounts of each transiently expressed HA antigen were loaded and separated by SDS–polyacrylamide gel electrophoresis (SDS-PAGE) under denaturing conditions, then transferred to PVDF membranes. After overnight blocking at 4 °C in blocking buffer (5% skim milk in PBST), membranes were incubated with H7 HA monoclonal antibody (prepared in our laboratory) at a 1:1000 dilution for 2 h, followed by washing. Membranes were then incubated with alkaline phosphatase-conjugated goat anti-rabbit IgG (Solarbio, Beijing, China) diluted at 1:5000 for 1 h. After further washing, the signal was detected with a chemiluminescent substrate kit. Protein imprinting was analysed for grayscale using ImageJ software.

### The construction of recombinant* S.* Typhimurium delivering expression plasmid for the HA antigen

The eukaryotic plasmids expressing the HA antigen (pS0017-9S7M, pS0017-CDS and pS0017-tPA-9S7M) were introduced into the *S.* Typhimurium rSC0130 competent cells prepared in our laboratory. These were plated on Luria–Bertani (LB) agar plates containing 0.2% arabinose without antibiotics and incubated overnight [25]. Suspected positive colonies were picked and identified using pS0017 identification primers (Forward: 5'- CGGTGGGAGGTCTATATAAGCAG-3', Reverse: 5'- CTGCATTCTAGTTGTGGTTTGTCC-3'). Clones with correct identification were designated as positive clones and named rSC0130(pS0017-9S7M), rSC0130(pS0017-CDS), and rSC0130(pS0017-tPA-9S7M), respectively.

### Identification of HA protein delivered by recombinant* S.* Typhimurium

HEK293T cells were infected with *S.* Typhimurium strains rSC0130(pS0017-9S7M), rSC0130(pS0017-CDS), and rSC0130(pS0017-tPA-9S7M). The bacterial strains were grown overnight at 37 °C, then subcultured to an OD_600_ of 0.8–0.9. Cultures were centrifuged and resuspended in phosphate-buffered saline (PBS). Confluent cells were infected with *S.* Typhimurium strains at a multiplicity of infection (MOI) of 40, with uninfected cells used as controls. After 1 h, monolayers were washed with PBS and treated with gentamicin (100 µg/mL) for 2 h to remove extracellular bacteria. Cells were then incubated for a further 48 h, harvested in 150 μL RIPA (radioimmunoprecipitation assay) lysis buffer (Solarbio, Beijing, China), and subjected to 2–4 cycles of sonication (5 s pulses with 60 s interval, at 50% amplitude). Supernatants were analysed for HA antigen expression by western blot. Grayscale analysis of the protein blot was performed using ImageJ software.

### Animal immunisation

To evaluate the immune response induced by rSC0130(pS0017-9S7M), 56 one-day-old SPF chickens (purchased from Boehringer Ingelheim Animal Health Products Co., Ltd., Beijing, China) were randomly assigned to four groups (*n* = 14 per group). On day 1, chickens in the rSC0130(pS0017-9S7M), rSC0130(pS0017-CDS), and rSC0130(pS0017) groups were orally immunised with 5 × 10^9^ CFU per bird. The PBS-immunised control group received oral immunisation with PBS on day 0. All experimental groups then received booster immunisations on days 14 and 28 with the same dose and route. Blood serum samples were collected from all SPF chicken groups on days 14, 28, and 42 for subsequent experiments.

To assess the immune response induced by rSC0130(pS0017-tPA-9S7M), 56 one-day-old SPF chickens were randomly allocated to four groups (*n* = 14 per group). The rSC0130(pS0017-tPA-9S7M), rSC0130(pS0017-9S7M), rSC0130(pS0017) and PBS-immunised groups were immunised following the same immunisation schedule as described above.

All the animal experiments were performed in triplicate.

### Haemagglutination-inhibition assay

The haemagglutination-inhibition (HI) assay was performed according to standard protocols. Briefly, 0.5% (v/v) avian red blood cells, 4 HA units of homologous H7N9 AIV, and specific sera pre-treated with receptor-destroying enzyme (Denka Seiken, Japan) at 37 ℃ for 18 h were used. The HI titre was defined as the highest serum dilution capable of inhibiting haemagglutination [35].

### Microneutralization (MN) assay

The microneutralisation (MN) assay was performed by incubating AIV (A/Chicken/China/QHD1/2019(H7N9)) at 100 × TCID_50_ with an equal volume of tenfold diluted specific heat-inactivated serum overnight at 37 °C in a humidified atmosphere containing 5% CO_2_. After incubation, 100 μL of the virus–serum mixture was added to a 96-well plate containing a monolayer of Madin–Darby canine kidney (MDCK) cells and incubated for 72 h at 37 °C, 5% CO_2_ [35]. Following this, the culture supernatant was mixed with an equal volume of 1% (v/v) chicken erythrocytes to assess haemagglutination. The MN titre was defined as the highest serum dilution showing an absence of haemagglutination.

### Lymphocyte proliferation assay

The lymphocyte proliferation was assessed using the CCK-8 assay (Solarbio, Beijing, China) [36]. Peripheral blood mononuclear cells (PBMCs) were isolated from samples collected 7 days after the third immunisation using a chicken PBMC isolation kit (Solarbio, Beijing, China). PBMCs were cultured in RPMI 1640 medium at a density of 2 × 10^5^ cells per well in a 96-well plate and maintained in a CO_2_ incubator at 37 °C for 48 h. During the incubation, cells were stimulated with β-propiolactone-inactivated H7N9 AIV or Concanavalin A (ConA). The inactivated virus was diluted to 10 μg/mL in RPMI 1640 medium, and ConA was diluted to 5 μg/mL, then added to the PBMCs. Untreated cells served as a blank control. After 48 h, 10 μL of CCK-8 reagent was added to each well, and the plates were further incubated for 2 h at 37 °C. Optical density (OD) was measured at 450 nm using an automated microplate reader. The stimulation index (SI) was calculated as the ratio of the OD of antigen-stimulated cells to that of unstimulated cells.

### The investigation of cytokine gene expression

PBMCs were isolated from blood samples collected 7 days after the third immunisation using a chicken PBMC kit (Solarbio, Beijing, China). Cells were cultured at 1 × 10^7^ cells per well in a 24-well plate and incubated at 37 °C with 5% CO_2_ for 48 h. They were stimulated with the same concentration of inactivated virus or ConA as described above. The mRNA expression levels of IFN-γ and IL-4 in PBMC samples were assessed by quantitative reverse transcription polymerase chain reaction (qRT-PCR). Total RNA was extracted using the Quick-cDNA Rapid Extraction kit (Vazyme, Nanjing, China). The purified RNA was reverse-transcribed into cDNA using PrimeScript™ RT Master Mix (TaKaRa, Beijing, China). qPCR was performed using Taq Pro Universal SYBR qPCR Master Mix (Vazyme, Nanjing, China) [34]. The qPCR protocol consisted of an initial denaturation at 95 °C for 30 s, followed by 40 cycles of denaturation at 95 °C for 10 s and 60 °C for 30 s. The primers are listed in Table 2. Gene expression levels were analysed using the 2^−ΔΔct^ method, with *β-actin* as the housekeeping gene.

**Table 2:** **Primer information in this study**

Name	Sequences (5'–3')	Reference
pS0017-F	CGGTGGGAGGTCTATATAAGCAG	This study
pS0017-R	CTGCATTCTAGTTGTGGTTTGTCC	
tPA	ATGGATGCAATGAAGAGAGGGCTCTGCTGTGTGCTGCTGCTGTGTGGAGCAGTCTTCGTTTCGCCCAGC	
IFN-γ-F	AGCTGACGGTGGACCTATTATT	[44]
IFN-γ-R	GGCTTTGCGCTGGATTC	
IL-4-F	TGAATGACATCCAGGGAGAG	
IL-4-R	GGCTTTGCATAAGAGCTCAG	
β-actin-F	CAACACAGTGCTGTCTGGTGG	
β-actin-R	ATCGTACTCCTGCTTGCTGATCC	

### Viral challenge

All groups of chickens were challenged with H7N9 AIV at 100 × LD_50_ on 42 days after the first immunisation. Clinical symptoms and mortality were observed in chickens for 12 days post-challenge. The Mantel–Cox method log-rank test was used to compare Kaplan–Meier survival curves for analysis. For histopathological analysis, haematoxylin–eosin (HE) staining was conducted on lung tissues collected from chickens euthanised on day 48 or from those that died or were in poor condition before day 48, using the same method for lung tissue collection. Pulmonary pathological scores of each group were evaluated using a blinded histopathological evaluation protocol to quantify the severity of pulmonary lesions in chicken lung tissue, employing a 5-point ordinal scale. The grading criteria were defined as follows: 0, no obvious pathological changes; 1, congestion; 2, inflammatory infiltration; 3, inflammatory cell infiltration; 4, haemorrhage or necrosis; and 5, severe inflammatory cell infiltration. Higher numerical designations encompassed all pathological manifestations characteristic of preceding grades. Final scores were based on the most pronounced histopathological alterations identified through systematic microscopic examination [34]. All the animal experiments were performed in triplicate.

### Statistical analysis

All statistical analyses were based on at least three independent experiments. Statistical evaluations were conducted with one-way and two-way ANOVA, with differences considered significant when *P* < 0.05. GraphPad Prism was used for analysis, and data were presented as mean ± standard deviation (SD).

## Results

### H9 HA leader sequence can enhance the expression level of H7 HA protein in HEK293T cells

In our previous study, we observed that within identical eukaryotic expression plasmids, the expression level of HA protein from H9N2 AIV was significantly higher than that of HA protein from H7N9 AIV. Leader sequences are known to influence the immunogenicity and expression efficiency of HA [18], while non-coding regions play essential roles in RNA transcription and replication [37].

**Figure 1:**
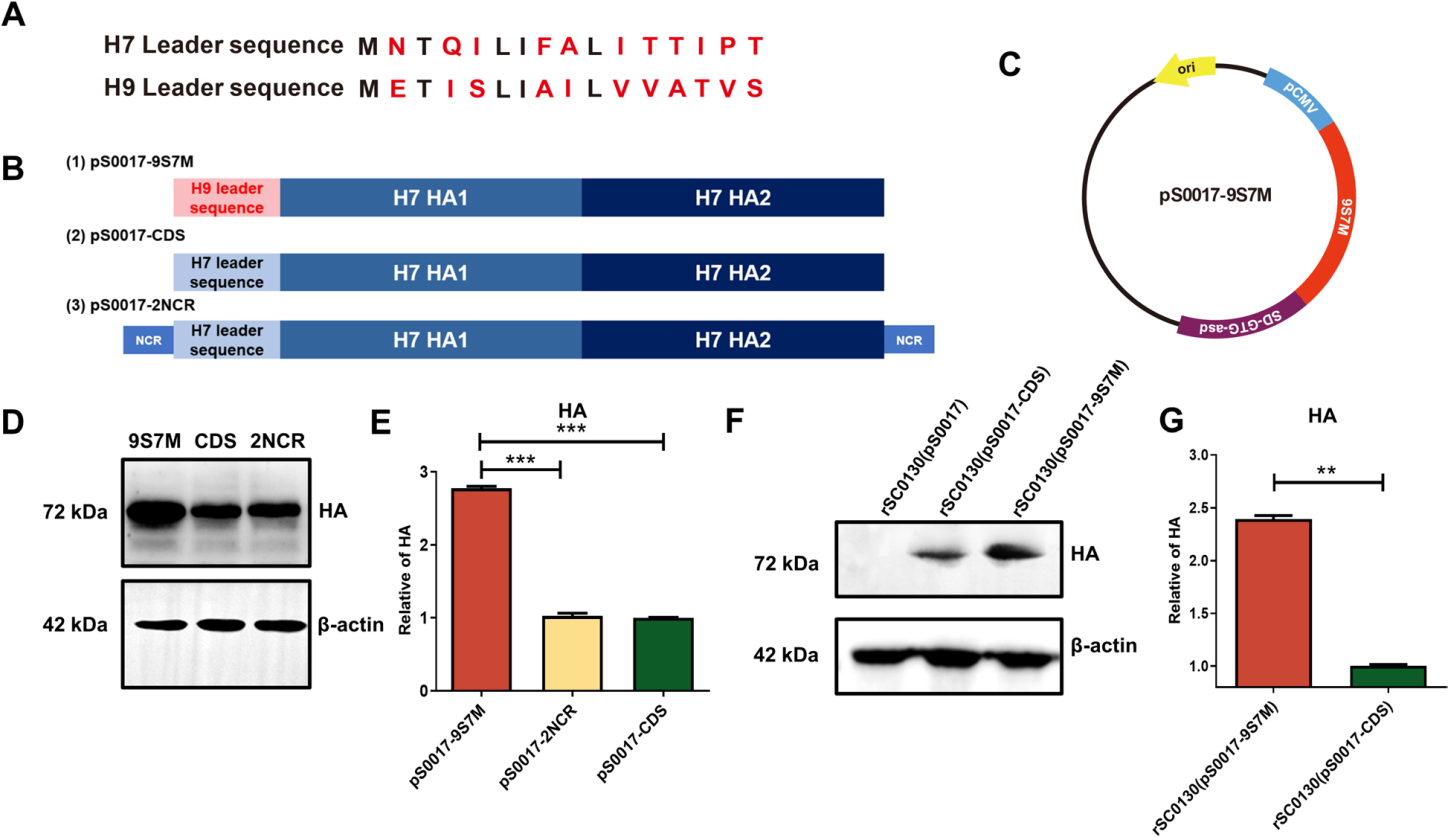
**Design and expression of H7 HA antigen. A** Alignment results of H7 HA and H9 HA leader sequences. **B** Schematic representation of the inserted fragment of type A influenza H7 HA gene in the recombinant eukaryotic expression plasmid pS0017, including the full-length HA antigen encoding region with the natural leader sequence of H7 HA (pS0017-CDS), the replacement of the natural leader sequence of H7 HA with the H9 HA leader sequence (pS0017-9S7M), and the HA antigen containing non-coding regions (pS0017-2NCR).** C** Schematic diagram of the recombinant eukaryotic expression plasmid pS0017-9S7M. **D** Expression of HA protein after transfection of HEK293T cells with pS0017-9S7M, pS0017-CDS, and pS0017-2NCR. **E** Grayscale analysis of the western blot results in **(D)** to compare the differences in expression levels. **F** Expression results of HA protein in candidate vaccine strains infected with HEK293T cells. **G** Grayscale analysis of the western blot results in **(F)** to compare the differences in expression levels. The data are expressed as the mean ± SD for each group (***P < 0.001, **P < 0.01, *P < 0.05, ns *P* > 0.05).

To compare the H9 and H7 HA leader sequences, we conducted amino acid sequence analysis, which revealed 11 amino acid differences (Figure 1A). To investigate the effects of the leader sequence and non-coding region on viral antigens expression from eukaryotic expression plasmids, we designed three types of H7 HA sequences: (1) a recombinant H7 HA sequence (9S7M) with the H9 HA leader sequence replacing the 1–48 bp leader sequence of H7 HA; (2) an H7 HA coding sequence containing only the coding region of H7 HA (used as a control); and (3) an H7 HA sequence (2NCR) containing both 5'and 3'non-coding regions (Figure 1B). These three HA sequences were codon-optimised for avian bias and SIRV system requirements in the attenuated *S*. Typhimurium vector, and were inserted into the eukaryotic expression plasmid pS0017, designated as pS0017-CDS, pS0017-9S7M, and pS0017-2NCR, respectively (Figure 1B and C).

After transient transfection of these plasmids into HEK293T cells, western blot revealed that the expression level of pS0017-9S7M was significantly higher than that of pS0017-2NCR and pS0017-CDS, respectively. In contrast, there was no difference between pS0017-2NCR and pS0017-CDS (Figure 1E). These outcomes suggest that the H9 HA leader sequence can enhance the expression level of the H7 HA protein.

### Characteristic of recombinant *S.* Typhimurium carrying H7 HA-9S7M protein with H9 HA leader sequence

Based on the previous experimental results, we selected the pS0017-9S7M plasmid, which exhibited the highest antigen expression level after transfection, together with the pS0017-CDS as a control, for transformation into recombinant *S.* Typhimurium strain rSC0130. This generated rSC0130(pS0017-9S7M) and rSC0130(pS0017-CDS), respectively. HEK293T cells were then infected with these strains, and protein expression of 9S7M and CDS in infected cells was detected by western blotting. Both rSC0130(pS0017-9S7M) and rSC0130(pS0017-CDS) were able to express HA using the cellular protein synthesis system after invasion of HEK293T cells (Figure 1F).

Moreover, the expression level of HA protein in rSC0130(pS0017-9S7M) was 2.4 times higher than in rSC0130(pS0017-CDS) (Figure 1G). These results indicate that the H9 leader sequence retains its ability to enhance expression of H7 HA-9S7M even after invasion of eukaryotic cells by recombinant attenuated *S.* Typhimurium.

### Immune responses induced by *S.* Typhimurium delivering H7 HA protein with H9 HA leader sequence

**Figure 2:**
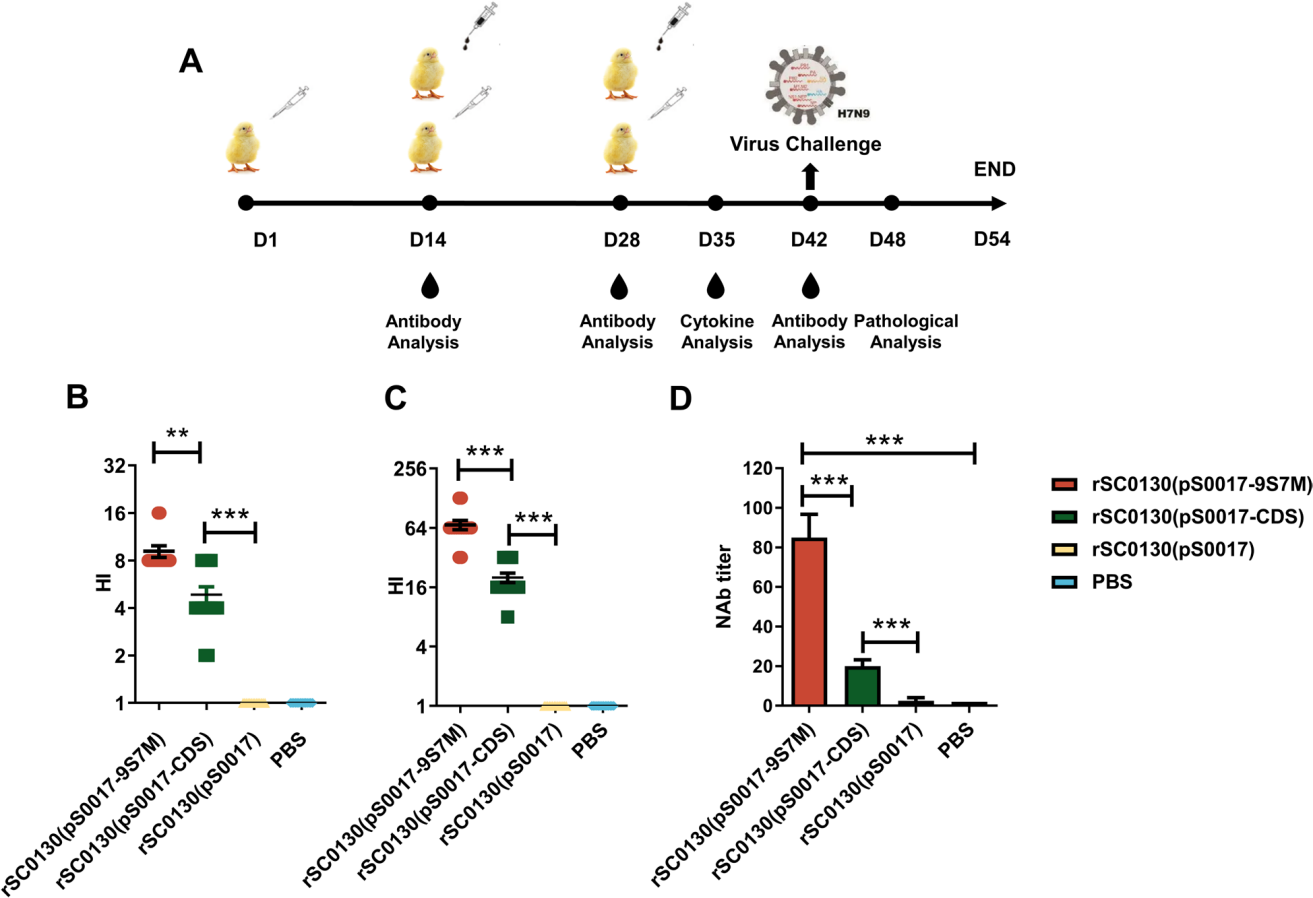
**Characteristics of humoral immunity induced by recombinant attenuated *S*. Typhimurium in immunised chickens. A** Immunisation schedule for all *Salmonella* vector vaccine groups.** B–C** Haemagglutination inhibition (HI) antibody titers at 28 **(B)** and 42 **(C)** days post-immunisation. **D** Microneutralization (MN) assay for neutralising antibody titers. The data are expressed as the mean ± SD for each group (***P < 0.001, **P < 0.01, *P < 0.05, ns *P* > 0.05).

To investigate the immune responses induced by recombinant attenuated *S.* Typhimurium strains rSC0130(pS0017-9S7M) and rSC0130(pS0017-CDS), SPF chickens were immunised as shown in Figure 2A. Serum samples were collected on days 14, 28, and 42 post-immunisation, and HI assays were performed to measure the humoral immune levels. No effective HI titres were detected after the first immunisation in any group. Following the second immunisation, rSC0130(pS0017-9S7M) induced significantly higher HI titres than rSC0130(pS0017-CDS) (Figure 2B).

After the third immunisation, HI titres induced by rSC0130(pS0017-9S7M) were again significantly higher, with a 3.4-fold difference compared to rSC0130(pS0017-CDS) (Figure 2C), consistent with the earlier trend. By day 42, the result of the serum neutralising capacity revealed that the neutralising titre induced by rSC0130(pS0017-9S7M) was significantly four times higher than that induced by rSC0130(pS0017-CDS) (Figure 2D). These results demonstrate that the H9 HA leader sequence enhances the humoral immune response induced by the candidate vaccine of recombinant attenuated *S.* Typhimurium delivering H7 HA protein.

**Figure 3:**
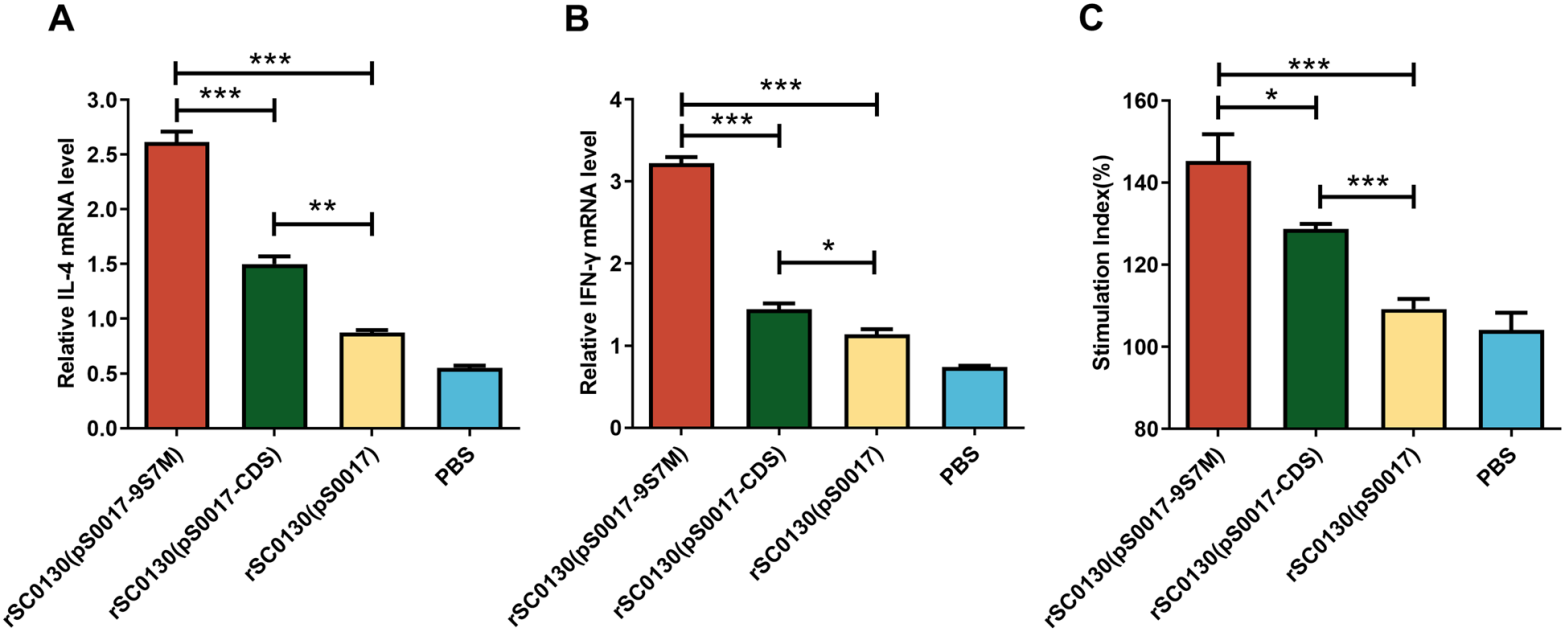
**Cellular immune characteristics induced by recombinant attenuated *S*. Typhimurium in immunised chickens. A–B** Relative mRNA levels of IL-4 and IFN-γ in peripheral blood mononuclear cells (PBMCs) of immunised chickens.** C** Proliferative response of PBMCs stimulated with H7N9 antigen post-immunisation. The data are expressed as the mean ± SD for each group (***P < 0.001, **P < 0.01, *P < 0.05, ns *P* > 0.05).

To investigate the cellular immune response induced by rSC0130(pS0017-9S7M) and rSC0130(pS0017-CDS), peripheral blood lymphocytes (PBLs) were isolated from all groups of chickens one week after the third immunisation (day 32). Expression levels of intracellular cytokines and lymphocyte proliferation capacity were then assessed. The vaccine-induced cellular immune response was evaluated by measuring the mRNA levels of IL-4 and IFN-γ using quantitative real-time PCR (qRT-PCR). The results showed that immunisation with rSC0130(pS0017-9S7M) significantly increased IL-4 mRNA levels after immunisation, which were 1.7-fold higher than in the rSC0130(pS0017-CDS) group (Figure 3A). Similarly, IFN-γ mRNA levels were significantly increased in the rSC0130(pS0017-9S7M) group compared to the rSC0130(pS0017-CDS) group, with a 2.1-fold rise observed (Figure 3B). These results indicate that the H9 HA leader sequence enhances the Th1- and Th2-mediated cellular immune responses induced by the recombinant attenuated *S.* Typhimurium-delivered H7 HA-9S7M protein vaccine. PBL proliferation was also measured using the CCK-8 assay, showing that, under the same inactivated virus stimulation, the proliferation capacity of lymphocytes from the rSC0130(pS0017-9S7M) immunisation group (Figure 3C) was significantly higher than that of the rSC0130(pS0017-CDS) group.

### *S.* Typhimurium delivering H7 HA protein with H9 HA leader sequence enhances protection against homologous virus challenge

**Figure 4:**
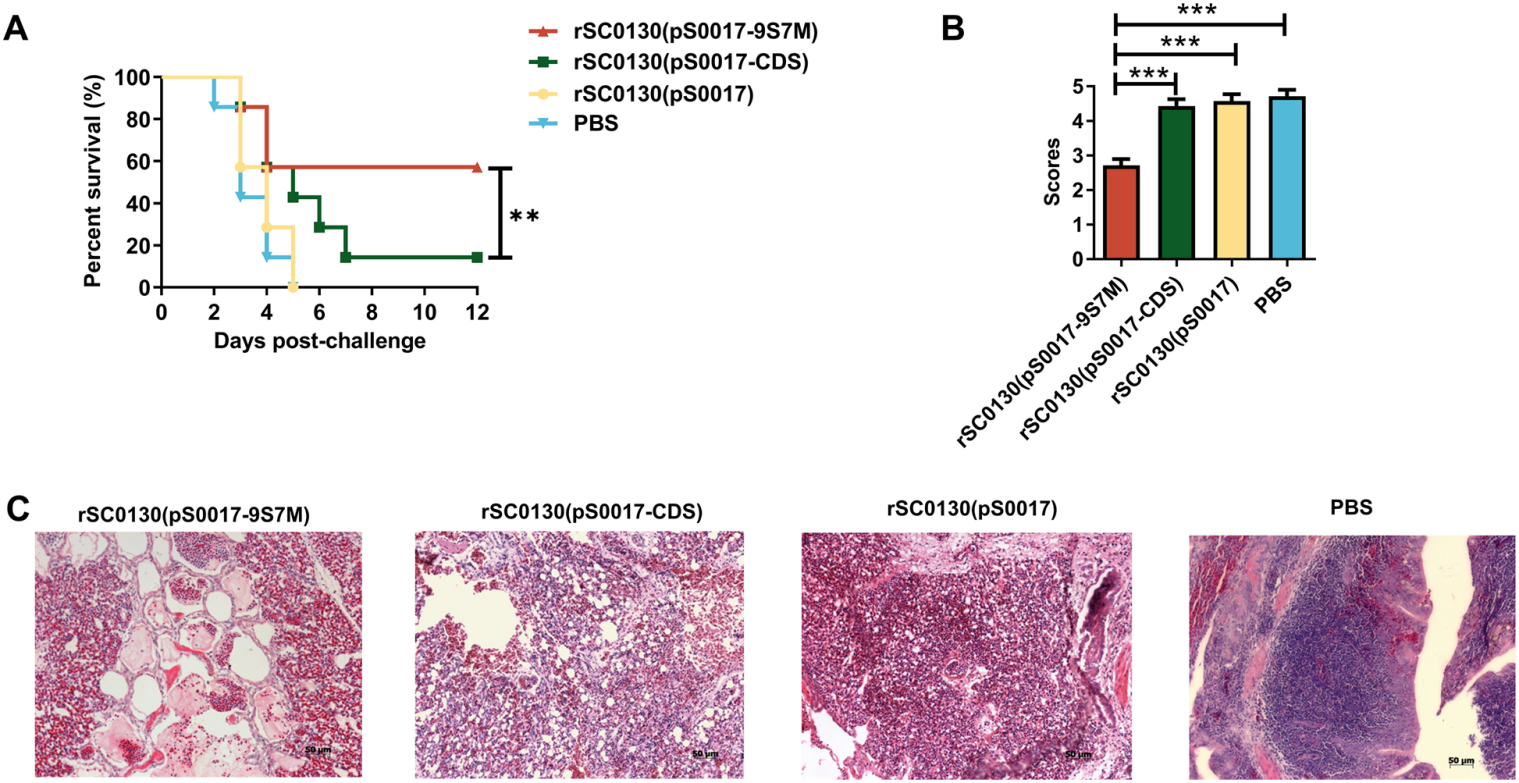
**Protective effect of immunity induced by recombinant attenuated *S*. Typhimurium against H7N9 avian influenza virus challenge. A** Immune protection efficacy of each candidate vaccine strain. Log-rank Mantel–Cox method for Kaplan–Meier survival curve analysis was used for curve comparison. **B** The pulmonary pathological scores of each immunisation group. **C** Pulmonary pathological lesions of rSC0130(pS0017-9S7M), rSC0130(pS0017-CDS), rSC0130(pS0017) and PBS-immunised group on the sixth day post-challenge. The data are expressed as the mean ± SD for each group (***P < 0.001, **P < 0.01, *P < 0.05, ns *P* > 0.05).

To evaluate the protective efficacy of immunisation with rSC0130(pS0017-9S7M) against homologous AIV challenge, chickens were challenged on day 42 with H7N9 AIV at 100 × LD_50_. Mortality was monitored for 12 days, and lung pathology was assessed on day 6 post-challenge. The mortality of all groups is shown in Figure 4A. Chickens immunised with rSC0130(pS0017-CDS) exhibited clinical symptoms (coughing, cyanosis of the comb, facial swelling, respiratory distress, etc.) from day 3 post-challenge, with most dead by day 7, resulting in a final protection rate of only 14%. In contrast, chickens in the rSC0130(pS0017-9S7M) group showed milder clinical symptoms and mortality, with only a few deaths occurring within 2–4 days, yielding a final protection rate of 57%. These results indicate that the H9 HA leader sequence can, to some extent, enhance the protective efficacy against homologous AIV challenge induced by the recombinant attenuated *S.* Typhimurium-delivered H7 HA-9S7M protein vaccine.

To examine lung pathology changes in SPF chickens immunised with rSC0130(pS0017-9S7M) after virus challenge, chickens showing severe symptoms during the first six days and additional birds on day 6 were euthanised for lung tissue collection to prepare pathological sections for microscopic observation and pathological scoring (Figure 4B and C). HE staining revealed that chickens in the rSC0130(pS0017-9S7M) group had milder pathological changes in the lung tissue, characterised by moderate infiltration of inflammatory cells, congestion, and exudation in the alveolar cavities, while the alveolar structure remained relatively intact. In contrast, chickens in the rSC0130(pS0017-CDS) group exhibited severe interstitial pneumonia, diffuse infiltration of inflammatory cells, extensive haemorrhagic pneumonia, and destruction of the alveolar structure. Chickens in the rSC0130(pS0017) and PBS groups also showed severe interstitial pneumonia, characterised by severe infiltration of inflammatory cells in the alveolar cavities, extensive haemorrhagic pneumonia, destruction of the alveolar structure, with necrotic structures observed in the PBS group. These findings suggest that the H9 HA leader sequence can mitigate lung pathology to some extent, following virus challenge in chickens immunised with the recombinant attenuated *S.* Typhimurium vaccine delivering H7 HA.

### tPA signal sequence can augment the expression levels of H7 HA protein

**Figure 5:**
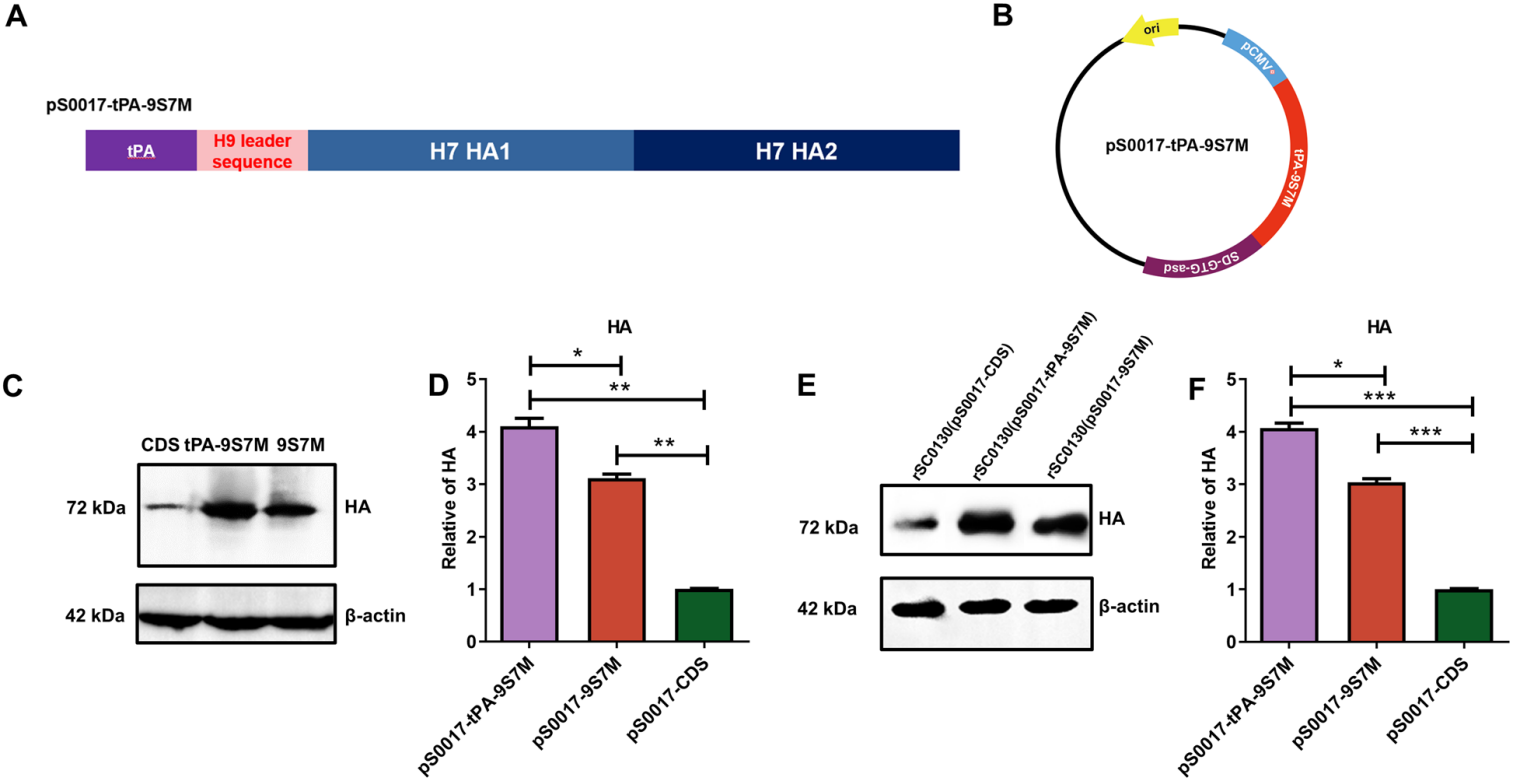
**Design and expression of tPA-9S7M. A** Schematic representation of the inserted fragment of type A influenza H7 HA gene in the recombinant eukaryotic expression plasmid pS0017, including the 9S7M fragment with the tPA signal sequence added (pS0017-tPA-9S7M). **B** Schematic diagram of the recombinant eukaryotic expression plasmid pS0017-tPA-9S7M.** C** Expression of HA protein after transfection of HEK292t cells with pS0017-9S7M, pS0017-tPA-9S7M, and pS0017-CDS. **D** Grayscale analysis of the western blot results in **C** to compare the differences in expression levels. **E** Expression results of HA protein in candidate vaccine strains infected with HEK293T cells. **F** Grayscale analysis of the western blot results in **(E)** to compare the differences in expression levels. The data are expressed as the mean ± SD for each group (***P < 0.001, **P < 0.01, *P < 0.05, ns *P* > 0.05).

Studies have shown that the tPA signal sequence can effectively increase antigen expression and secretion. To boost H7 HA antigen expression, we engineered tPA-9S7M by fusing the tPA signal sequence to the 5' terminus of the 9S7M construct (Figure 5A). This design was cloned into the plasmid pS0017 (Figure 5B) and transformed into *S.* Typhimurium strain rSC0130.

Following transfection into HEK293T cells, cell lysates were collected and analysed by western blot (Figure 5C). The expression level of pS0017-tPA-9S7M was 1.25 times higher than that of pS0017-9S7M, showing a significant difference (Figure 5D). In *S.* Typhimurium-infected HEK293T cells, rSC0130(pS0017-tPA-9S7M) showed 1.23-fold greater HA expression than rSC0130(pS0017-9S7M) (Figure 5E and F). These results indicate that incorporating the tPA signal sequence together with the H9 leader sequence can enhance H7 HA protein expression.

### tPA signal sequence can enhance the antibody levels induced by *S.* Typhimurium delivering the H7 HA protein candidate

**Figure 6:**
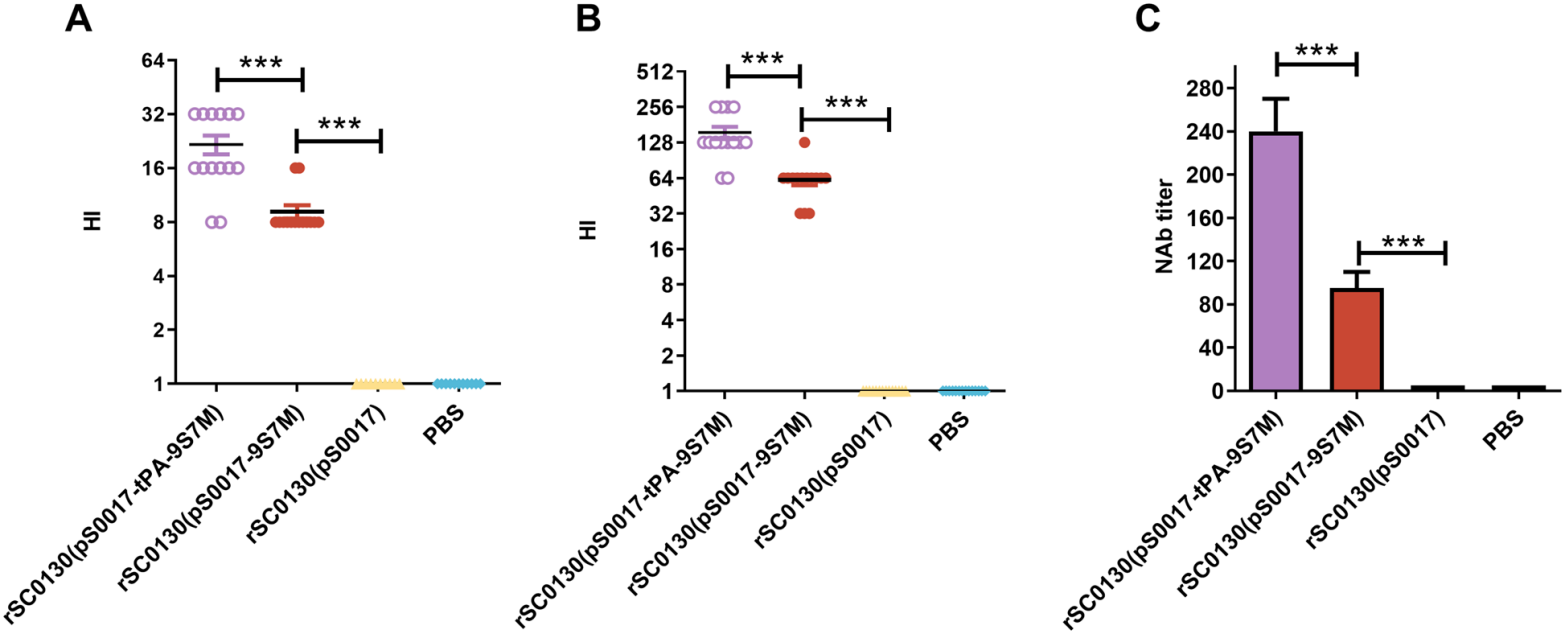
**Characteristics of humoral immunity induced by rSC0130(pS0017-tPA-9S7M) in immunised chickens. A–B** Haemagglutination inhibition (HI) antibody titers at 28 **(A)** and 42 **(B)** days post-immunisation. **C** Microneutralization (MN) assay for neutralising antibody titers. The data are expressed as the mean ± SD for each group (***P < 0.001, **P < 0.01, *P < 0.05, ns *P* > 0.05).

To evaluate humoral immunity, SPF chickens were immunised with recombinant *S.* Typhimurium strains rSC0130(pS0017-tPA-9S7M) and rSC0130(pS0017-9S7M). After the second immunisation, rSC0130(pS0017-tPA-9S7M) induced an HI titre of 2^4.4^, while rSC0130(pS0017-9S7M) reached 2^3.2^ (Figure 6A). Following the third immunisation, rSC0130(pS0017-tPA-9S7M) elicited a significantly higher HI titre of 2^7.3^ compared to 2^5.9^ in the rSC0130(pS0017-9S7M) group (Figure 6B). Microneutralisation (MN) assays further confirmed a 2.5-fold higher neutralising antibody titre in the rSC0130(pS0017-tPA-9S7M) group (Figure 6C). Collectively, these results suggest that the recombinant *S.* Typhimurium delivering H7 HA protein containing the tPA signal sequence elicits a stronger humoral immune response.

### tPA signal sequence improves the immune response induced by *S.* Typhimurium delivering H7 HA protein candidate vaccines

**Figure 7:**
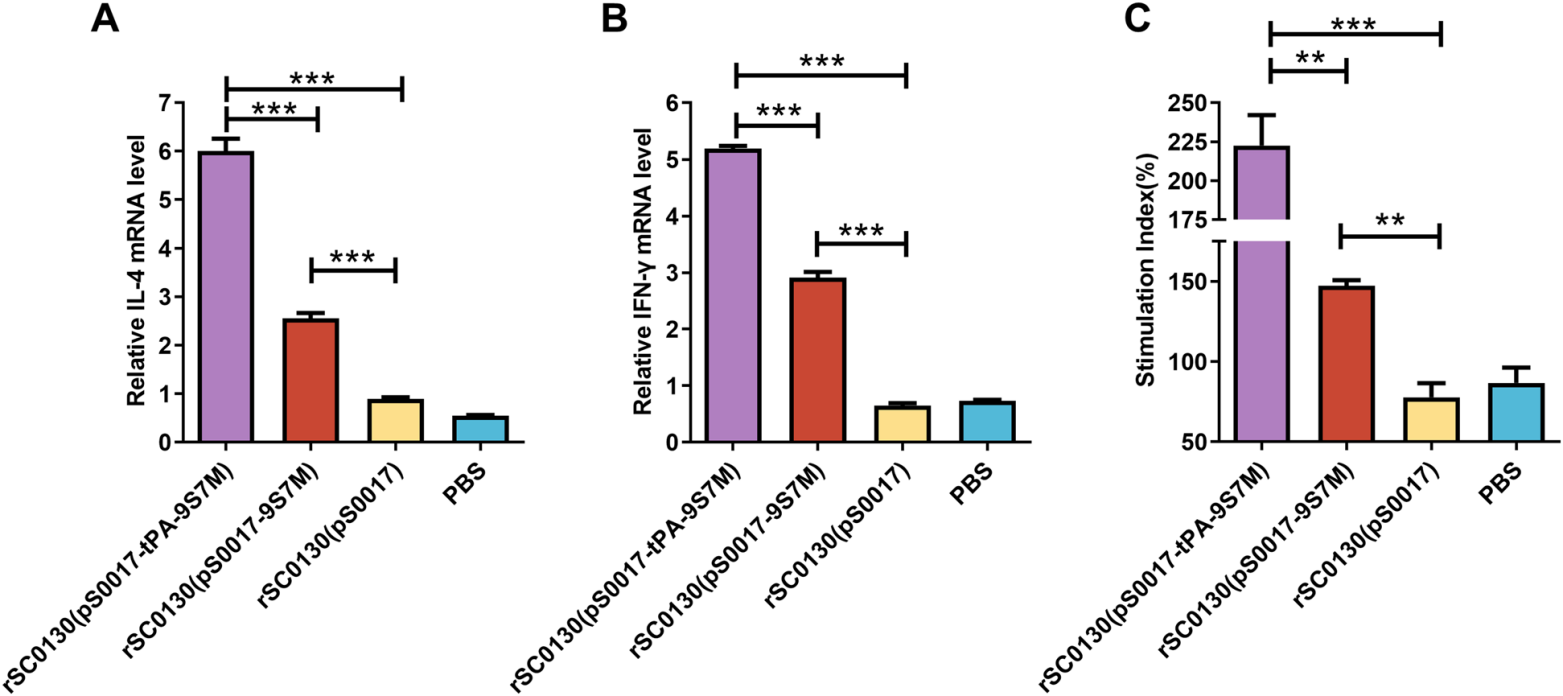
**Immune responses characteristics induced by rSC0130(pS0017-tPA-9S7M) in immunised chickens. A–B** Relative mRNA levels of IL-4 and IFN-γ in PBMCs of immunised chickens.** C** Proliferative response of PBMCs stimulated with H7N9 antigen post-immunisation. The data are expressed as the mean ± SD for each group (***P < 0.001, **P < 0.01, *P < 0.05, ns *P* > 0.05).

To investigate the immune response elicited by rSC0130(pS0017-tPA-9S7M), PBMCs were isolated from all groups one week after the third immunisation. After antigen stimulation, cytokine expression levels and lymphocyte proliferation were assessed in SPF chickens. Compared to rSC0130(pS0017-9S7M), rSC0130(pS0017-tPA-9S7M) significantly up-regulated Th2- and Th1-associated cytokines, with 2.4-fold higher IL-4 and 1.7-fold increased IFN-γ mRNA levels in antigen-stimulated assays (Figure 7A and B). Lymphocyte proliferation in the rSC0130(pS0017-tPA-9S7M) group was also significantly higher than in the rSC0130(pS0017-9S7M) group (Figure 7C). These findings suggest that incorporating the tPA signal sequence alongside the H9 HA leader sequence can enhance the cellular immune response induced by the recombinant *S.* Typhimurium vector delivering the H7 HA protein vaccine.

### tPA signal sequence enhances protection against homologous virus challenge

**Figure 8:**
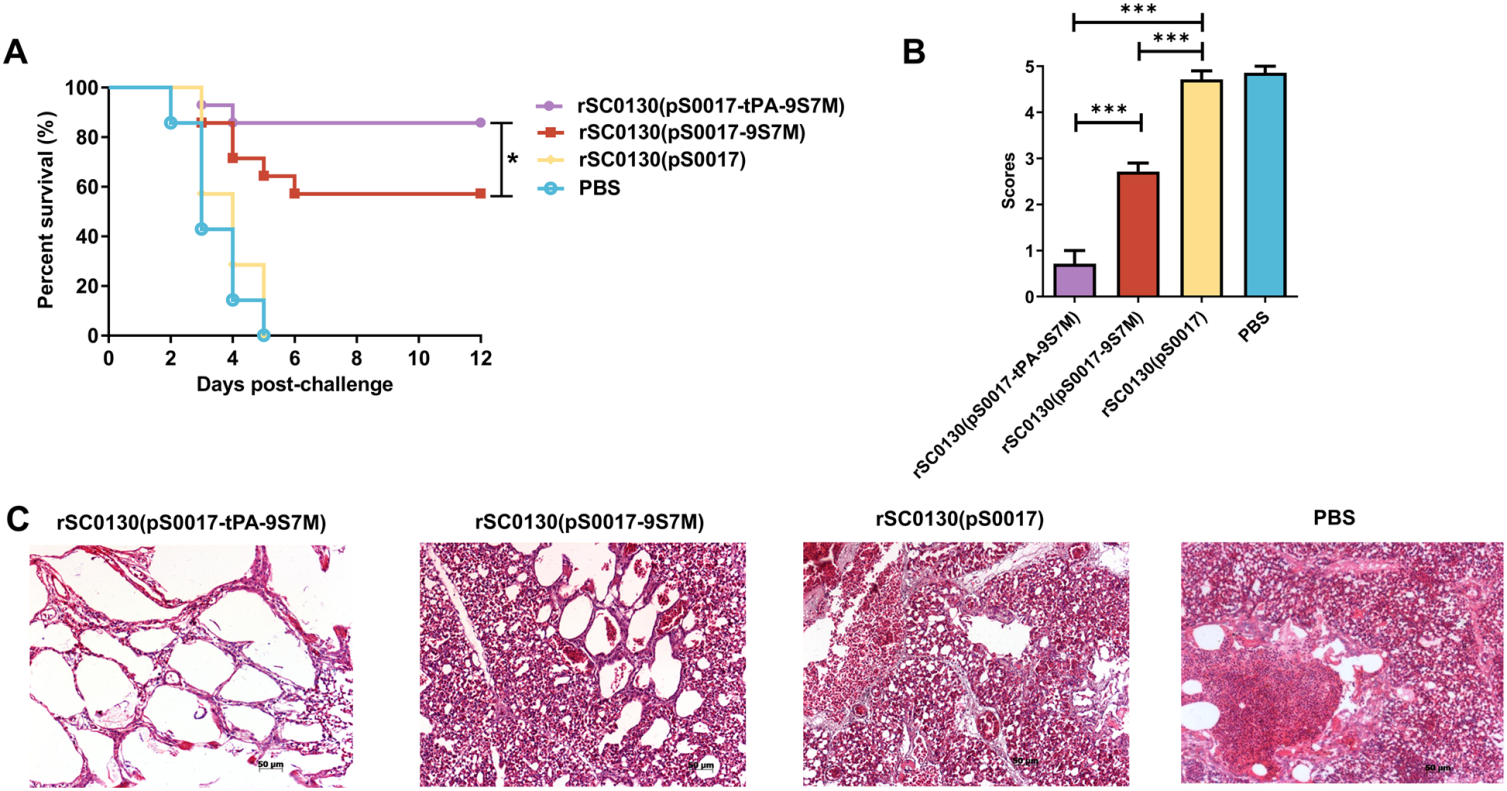
**Protective effect of immunity induced by rSC0130(pS0017-tPA-9S7M) against H7N9 avian influenza virus challenge. A** Immune protection efficacy of each candidate vaccine strain. **B** Pulmonary pathological scores of each immunisation group. **C** Pulmonary pathological lesions of rSC0130(pS0017-tPA-9S7M), rSC0130(pS0017-9S7M), rSC0130(pS0017) and PBS-immunised group on the sixth day post-challenge. The data are expressed as the mean ± SD for each group (***P < 0.001, **P < 0.01, *P < 0.05, ns *P* > 0.05).

To evaluate the protective efficacy of rSC0130(pS0017-tPA-9S7M) against homologous virus challenge, chickens were challenged with H7N9 AIV at 100 × LD_50_ on 42 days after the first immunisation. Chickens immunised with rSC0130(pS0017-tPA-9S7M) showed 85.6% survival (Figure 8A), significantly higher than the 57% protection observed in the rSC0130(pS0017-9S7M) immunised group, along with reduced clinical severity and delayed mortality (days 2–4 post-challenge). These results indicate that the recombinant *S.* Typhimurium delivering H7 HA protein containing the tPA signal sequence significantly enhances protection against homologous virus challenge.

To detect the lung pathology changes in SPF chickens immunised with rSC0130(pS0017-tPA-9S7M) after virus challenge, chickens showing severe symptoms during the first six days and additional birds on day 6 were euthanised, and lung tissues were collected to prepare pathological sections for microscopic observation and pathological scoring (Figure 8B and C). HE staining revealed that the rSC0130(pS0017-tPA-9S7M) group showed no significant pathological changes in the lungs post-challenge with homologous H7N9 AIV, with alveolar structures remaining clear and intact. In contrast, the rSC0130(pS0017-9S7M) group exhibited pulmonary congestion and moderate inflammatory cell infiltration within the alveolar spaces. The rSC0130(pS0017) and PBS groups displayed similar structures, with extensive haemorrhage and inflammatory cell infiltration observed in the lungs post-challenge. These findings indicate that the recombinant *S.* Typhimurium delivering H7 HA protein containing the tPA signal sequence can mitigate lung pathological changes following homologous virus challenge.

## Discussion

Optimised heterologous leader sequences can significantly enhance target protein expression efficiency [38]. The H9N2 HA leader sequence mediates greater HA protein expression than the native leader sequence (Figure 1D). This result demonstrates that the H9 HA leader sequence can enhance HA protein expression levels. The enhanced transmission capacity recently observed in H9N2 AIV strains may partially account for the differential expression efficiency of HA proteins [39, 40]. Future studies on the structural dynamics of the HA protein could provide deeper insights into this phenomenon. In the present study, our findings highlight the crucial role of H9N2 HA leader sequences in enhancing heterologous HA expression levels‌, providing novel design principles for HA-based vaccine candidates‌.

The efficiency with which vectors or adjuvants deliver antigens or cargos into host cells directly determines the immunogenicity of the vaccine candidates [22, 27, 33]. In this study, *Salmonella* vector carrying the HA antigen mediated the expression of HA protein in host cells following direct infection (Figure 1F). These data strongly confirm that the strain incorporating the SIRV system can deliver exogenous plasmids into host cells and enable efficient heterologous expression. This, in turn, translates into high immunogenicity in vivo, characterised by robust cellular and humoral immune responses, as well as neutralising antibody responses (Figures 2 and 3), ultimately resulting in improved protection (Figure 4). The SIRV system has already been shown to enhance the intracellular delivery and release of heterologous cargo, an effect previously confirmed with the delivery of bacterial antigens, which are of prokaryotic origin [33]. By introducing eukaryotic antigens, we have now demonstrated that the *Salmonella* vector with the SIRV system can also efficiently deliver antigens of eukaryotic origin, laying the groundwork for other delivery platforms based on eukaryotic antigens.

Enhancing plasmid expression levels within cells is a critical factor in optimising *Salmonella*-based vaccines. Modifying constructs with signal peptides can strengthen the protective cellular immune response of vaccines [41]. In this study, we combined the *S.* Typhimurium vector rSC0130 with the H9 HA leader sequence, and found that the recombinant attenuated* S*. Typhimurium vaccine candidate rSC0130(pS0017-9S7M) induced higher levels of lymphocyte proliferation (Figure 3C) and IFN-γ transcription (Figure 3B). This finding demonstrates that the H9 HA leader sequence can enhance the cellular immune response induced by the recombinant attenuated *S.* Typhimurium delivering H7 HA protein vaccine. This outcome may help explain why rSC0130(pS0017-9S7M) outperforms rSC0130(pS0017-CDS) in challenge protection experiments.

Coupling the tPA signal sequence with exogenous antigens can significantly enhance the expression and secretion efficiency of antigen proteins in the host, thereby improving the immunogenicity of DNA vaccines [42, 43]. Consistently, we observed that the tPA-fused HA leader sequence from H9 enhanced protein expression levels in both plasmid transfection and bacterial delivery systems. Additionally, this tPA-fused HA leader sequence from H9 further elevated the specific antibody and neutralising antibody responses against the H7 AIV HA antigen (Figure 6A and C), and induced higher levels of IFN-γ and IL-4 expression (Figure 7A and B). In immunoprotection experiments, the rSC0130(pS0017-tPA-9S7M) group achieved improved survival rates, reaching 85.6%, which surpassed those of the rSC0130(pS0017-9S7M) group, and also showed reduced pulmonary pathological changes (Figure 8A–F). These findings demonstrate that the tPA-fused HA leader sequence from H9 can further enhance the protective efficacy of the recombinant *Salmonella* candidate vaccine in delivering H7 AIV HA antigens.

In summary, we have integrated the H9N2 AIV HA leader sequence, the tPA signal peptide, and the SIRV system, which mediates antigen delivery and release, into the novel recombinant vector rSC0130(pS0017-9S7M).

This novel vector enhances the heterologous expression of the HA antigen protein and elicits stronger systemic immune responses. The rSC0130(pS0017-9S7M) strain also provides effective protection against a lethal dose challenge of H7N9 AIV in vivo, offering new perspectives for developing a *Salmonella* live vector to deliver viral antigens.

## Data Availability

All data generated or analysed during this study are presented in the article, and materials are available from the corresponding author upon reasonable request.
